# Observation, laser photocoagulation or anti-VEGF therapy in the management of retinal arterial macroaneurysms

**DOI:** 10.1186/s12886-022-02641-2

**Published:** 2022-11-02

**Authors:** Yuelin Wang, Hong Du, Xinyu Zhao, Lihui Meng, Youxin Chen

**Affiliations:** 1 Department of Ophthalmology, Peking Union Medical College Hospital, Chinese Academy of Medical Sciences, Beijing, 100730 China; 2 Key Lab of Ocular Fundus Disease, Chinese Academy of Medical Sciences, Beijing, 100730 China

**Keywords:** Retinal arterial macroaneurysm, Management, Clinical characteristics, Visual acuity, Central retinal thickness

## Abstract

**Background:**

To explore the efficacy of observation, laser photocoagulation, and anti-VEGF in the management of retinal arterial macroaneurysm (RAM).

**Methods:**

We retrospectively included patients diagnosed with RAM at the Peking Union Medical College Hospital (PUMCH) from 2003 to 2021, and comprehensively reviewed cases documented in the literature from multiple databases (PROSPERO protocol CRD42022310417). Patients were categorized into 3 groups: the observation group, anti-VEGF group, and laser photocoagulation group. LogMAR visual acuity (VA) and central retinal thickness (CMT) at the end of the follow-up were analyzed.

**Results:**

A total of 14 patients from the PUMCH and 210 patients from the literature review were included. VA and CMT in patients who underwent observation, laser photocoagulation, and anti-VEGF therapies were significantly improved from baseline (*p* < 0.05), with changes in LogMAR VA improved by -0.34 ± 0.68, -0.17 ± 0.58, and -0.45 ± 0.62 and changes in CMT improved by -148.26 ± 138.99 µm, -185.61 ± 130.37 µm, and -287.45 ± 171.87 µm, respectively. Subgroup analysis revealed that anti-VEGF therapy was used in patients with worse VA than patients who underwent laser photocoagulation (*p* = 0.010), but achieved better improvement in VA than the laser photocoagulation group (*p* = 0.049). Patients treated with anti-VEGF also had thicker CMT than the observation group (*p* = 0.013), and experienced better changes in CMT than the observation, as well as laser photocoagulation groups (*p* = 0.005; *p* = 0.047).

**Conclusion:**

Observation, anti-VEGF, and laser photocoagulation are effective therapeutic methods for the management of RAM, and anti-VEGF therapy is intended to better improve patients with severe VA and CMT.

**Supplementary Information:**

The online version contains supplementary material available at 10.1186/s12886-022-02641-2.

## Background

Retinal arterial macroaneurysm (RAM) refers to the localized fusiform or saccular dilatation of a retinal arterial vessel within the first three bifurcations, which was first described by Robertson in 1973 [1]. The prevalence of RAM ranges from 0.01% to 0.07%, which means that it is rare and easily misdiagnosed [2, 3]. RAM rupture with retinal hemorrhage, exudation, edema, or vitreous hemorrhage can sometimes cause severe vision loss. If the macula is involved, impaired vision can occur. Active RAM is classified into hemorrhagic or exudative types depending on whether hemorrhage or exudates are the major factor, measure more than 1 disc diameter and are responsible for visual loss [4].

Currently, there are no standard treatment guidelines for RAM. The proposed management options for RAM include observation, laser photocoagulation, anti-VEGF therapy, and surgical operations. Previous studies [5–7] suggested that in most cases, RAM is self-healing, and nonsurgical management is often considered to be the preferred treatment for RAM, as it does not depend on technical limitation. However, the results of different modes of management of RAM vary.

The aim of this study was to retrospectively analyze the nonsurgical clinical management of RAM and to evaluate the circumstances under which observation, laser photocoagulation, and anti-VEGF therapy should be used.

## Method

### Ethical compliance

This retrospective study was approved by the Institutional Review Board of Peking Union Medical College Hospital and conducted following the tenets of the Declaration of Helsinki. Written informed consent was provided to each patient before examination.

### Patient selection

Patients who underwent angiography at Peking Union Medical College Hospital (PUMCH) from May 2003 to January 2021 were retrospectively, consecutively selected. All patients were informed in detail of the purpose of the study and the complications of RAM management, and all signed an informed consent form before examination. RAM with (1) cyst-like or fusiform retinal aneurysm-like expansion observed on the fundus photograph, (2) local arterial expansion found by fundus fluorescein angiography (FFA) or indocyanine green angiography (ICGA), (3) patients underwent observation, laser photocoagulation, or anti-VEGF therapy, and (4) at least one follow-up with VA and OCT were recorded. Patients were excluded from this study if they (1) had other retinal diseases aside from RAM or (2) had undergone any ophthalmic management within the previous 6 months.

We searched PubMed, EMBASE, the Cochrane Central Register of Controlled Trials, the Cochrane Database of Systematic Reviews, CNKI (China National Knowledge Infrastructure), Web of Science, and the clinicaltrials.gov website from inception to Jan 1st, 2022, without language restrictions. The selected keywords were “retinal arterial macroaneurysm”, “observation”, “anti-VEGF”, and “laser photocoagulation”, among others. The inclusion requirements for articles were (1) Participant: patient diagnosed with RAM; (2) Intervention: observation, or a single session of laser photocoagulation, or anti-VEGF was conducted; (3) Comparison: at least one comparison existed in the study; (4) Outcomes: articles with sufficient and detailed data, including the description of individual primary VA, final VA; and/or primary CMT, final CMT. (5) Study type: comparative studies, including retrospective comparative studies and prospective studies. The following were the exclusion criteria: (1) RAM accompanied by other serious fundus diseases, such as Coats’ disease and vasculitis. (2) Cases that could not be accurately diagnosed. The detailed search strategy is shown in Additional file 1. The protocol for this systematic review is registered with PROSPERO CRD42022310417.

### Data extraction and statistical analysis

For RAM patients from the PUMCH, clinical characteristics and multimodal fundus imaging, including FFA, ICGA, and optical coherence tomography (OCT) of the disease, were analyzed. For included patients from the literature, the name of the author, year of publication, study design, and outcomes were extracted from each study. The primary outcomes were (1) VA [visual acuity was converted to the logarithm of minimum angle of resolution (logMAR) units for statistical analyses] from baseline to the last follow-up; (2) CMT detected by OCT from baseline to the last follow-up.

Continuous or discrete variables are presented as the means and standard deviations or counts (%). Comparisons of paired or unpaired data were made using Khi-2, Fisher or Wilcoxon for quantitative variables, and the paired or unpaired Student’s t test for normally distributed data, while nonparametric Wilcoxon Signed Ranks and Mann–Whitney U test were used for nonnormal data. One-way analysis of variance (ANOVA) test accompanied by LSD post hoc analysis or multigroup chi-square tests combined with Bonferroni post hoc correction were used for continuous or categorical variables among different treatments of PUMCH patients. Head-to-head studies were separately compared in the combined analysis of different treatment comparisons with pairwise comparison in case of large heterogeneity among studies. A p value < 0.05 was considered statistically significant. All statistical analyses were performed by SPSS 26.0 (SPSS Inc.; Chicago, IL, USA) or GraphPad Prism 8.0 (GraphPad Software Inc., San Diego, CA, USA) software.

## Results

### Cases in Peking Union Medical College Hospital

A total of 14 patients diagnosed with RAM were included. Table 1 summarizes the characteristics of RAM patients from the PUMCH. Among them, 11 were women (78.5%); the average age was 71.6 ± 6.5 years old. Ten patients had systemic hypertension under medical control. All the cases exhibited monocular single RAM with round or fusiform shape. Aneurysms were located in the temporal artery in 13 cases, accounting for 92.8%; 10 cases were located in the supratemporal artery. Aneurysms were located in the main trunk and 1^st^ and 2^nd^ branches in 3, 7, and 4 cases, accounting for 21.4%, 50.0%, and 28.5%, respectively. Of all the cases, 7 cases had different degrees of retinal hemorrhage (50.0%). Hemorrhage or edema involved the macula in 6 eyes. Among the included patients, 5 patients received observation, 4 patients received direct laser photocoagulation (around 532 nm green laser, about 200um in the diameter of the light spot around and/or direct to the aneurysm, 100–300 mW for laser power, 0.2 ~ 0.3 s for laser duration, and level III for laser response), and 5 patients underwent anti-VEGF therapy. All patients in laser photocoagulation or anti-VEGF group had not been previous treat by either anti-VEGF or laser photocoagulation therapies. One patient in the observation group had been treated with laser 4 months ago. No complications, such as vitreous hemorrhage, aneurysm rupture, were observed after laser or anti-VEGF treatment.

**Table 1:** Characteristics of RAM patients from PUMCH

	Observation (*n =* 5)	Laser Photocoagulation (*n =* 4)	Anti-VEGF (*n =* 5)	O. vs L.^a^	O. vs A.^a^	L. vs A.^a^
				*p*	*p*	*p*
Age(y)	68.20 ± 6.38	71.50 ± 6.46	75.20 ± 6.22	0.454	0.109	0.403
Sex(F)	2	4	5	0.501	0.501	1.000
Eye (OS)	5	2	2	0.501	0.501	1.000
Hypertension	4	2	2	1.000	1.000	1.000
Diabetes	2	0	2	1.000	1.000	1.000
Complication (Exudative)	2	4	1	0.501	1.000	0.143
No past treatment	4	4	5	1.000	1.000	1.000
Preretinal hemorrhage	1	0	4	1.000	0.618	0.143
Macular hard exudates	2	2	0	1.000	1.000	0.501
Distance from fovea	3098.80 ± 1712.84	2690.75 ± 874.44	1541.4 ± 1202.00	0.658	0.094	0.228
Duration of symptoms	3.60 ± 2.19	1.50 ± 0.58	2.20 ± 2.16	0.125	0.265	0.591
Interval(m)	7.40 ± 5.13	2.75 ± 1.50	5.20 ± 3.96	0.110	0.401	0.379
Initial LogMAR VA (mean VA in decimal)	0.18 ± 0.20 (0.16)	0.58 ± 0.33 (0.26)	1.16 ± 0.78 (0.07)	0.278	0.012**	0.119
Initial CMT (μm)	260.40 ± 89.98	377.50 ± 85.25	490.20 ± 210.50	0.254	0.029**	0.271

*VA* Visual acuity, *CMT*, Central macular thickness

^a^ *O* Observation, *L* Laser photocoagulation, *A* Anti-VEGF treatment

^**^P < 0.05

**Fig. 1:**
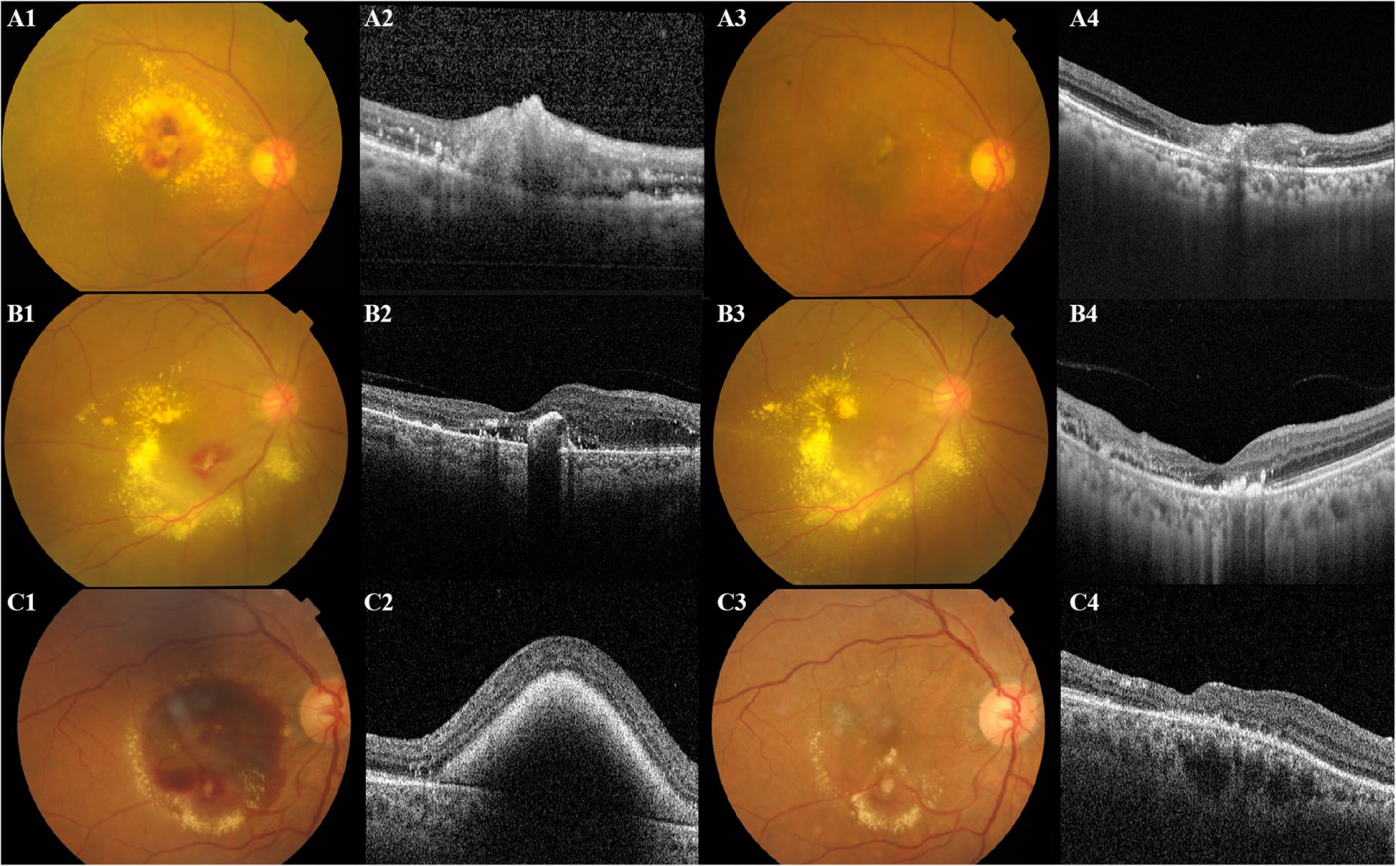
Examples of different managements of RAM in the PUMCH. **A** A 72-year-old female was diagnosed with RAM, with a VA of 0.5 (LogMAR) and CMT of 232 µm. After 1 month of observation, her final VA was 0.5 (LogMAR), and her CMT dropped to 191 µm. **B** A 74-year-old female patient had a VA of 0.2 (LogMAR) and CMT of 304 µm. She underwent laser photocoagulation directly to the RAM lesion and achieved a final VA of 0.2 and a CMT of 289 µm. **C** A 75-year-old female was diagnosed with RAM with a VA classified using Finger Count (2.3 of LogMAR) and CMT of 735 µm. After 4 months of anti-VEGF therapy, her VA improved to 1.3 (LogMAR) and CMT to 239 µm

Among these patients, 4 of 5 patients in the observation group had improved or remained the same VA, while all patients in laser photocoagulation group and anti-VEGF group were improved or remained unchanged VA; the CMT of all patients in PUMCH were decreased to varying degrees. Figure 1 provided examples of different treatments of RAM in PUMCH.

### Analysis of management of 224 RAM cases

**Fig. 2:**
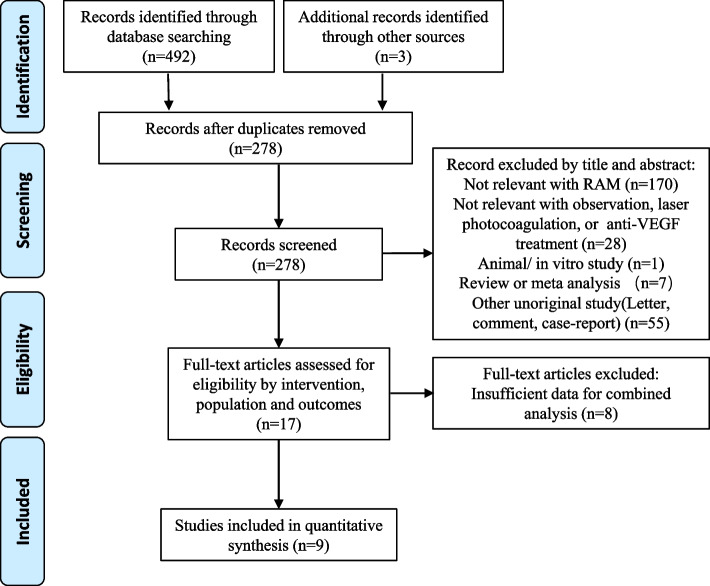
Flowchart of selection process in the comparison of compared studies of RAM non-surgical treatment

Initially, 492 records were identified through database searching, and 3 additional records were identified using other sources. After excluding 478 records by screening the titles and abstracts, a total of 17 manuscripts were fully examined (see Fig. 2). We ultimately included 9 studies for combined analysis (see Table 2). Of the included studies, 7 studies compared the efficacy of observation and laser photocoagulation, 1 study compared observation and anti-VEGF treatment, and 1 study included observation, anti-VEGF, and laser photocoagulation. Two studies reported the ratio of RAM patients who combined with diabetes mellitus (4.7% and 12.9%).

**Table 2:** Characteristics of included studies

Study	Year		N	Sex (Female, %)	Mean age	Treatment	N	Exudative RAM (%)	Mean Interval (m)	Baseline LogMAR VA (mean VA in decimal)	Baseline CMT (μm)
Palestine	1982		31	22(71)	68.0	Observation	25	18(72)	35.4	0.63 ± 0.71 (0.23)	NA
						Laser	6	6(100)	36.1	0.48 ± 0.07 (0.33)	NA
Abdel	1986		21	15(71)	68.7	Observation	10	4(40)	23.5	0.73 ± 0.49 (0.19)	NA
						Laser	11	9(82)	38.8	0.92 ± 0.75 (0.12)	NA
Brown	1994		42	NA	70.0	Observation	26	24(92)	39.5	1.08 ± 0.91 (0.08)	NA
						Laser	16	13(81)	42.9	1.08 ± 0.71 (0.08)	NA
Moosavi	2006		10	6(60)	71.0	Observation	9	5(56)	4.2	0.69 ± 0.86 (0.2)	NA
					81.0	Laser	1	1(100)	4	1 (0.1)	NA
Tsujikawa	2009		13	10(77)	74.3	Observation	2	2(100)	8	0.41 ± 0.16 (0.39)	418.00 ± 145.66
						Laser	11	6(55)	18	0.50 ± 0.27 (0.32)	438.55 ± 130.33
Cho	2013		23	19(82)	71.0	Observation	12	2(17)	10.5	1.36 ± 0.72 (0.04)	367.00 ± 105.68
						Anti-VEGF	11	4(36)	11.2	0.86 ± 0.56 (0.14)	466.73 ± 128.86
Koinzer	2015		31	22(71)	75.4	Observation	16	1(6)	31.3	0.48 ± 0.40 (0.33)	NA
						Laser	15	6(40)	37.2	0.55 ± 0.37 (0.28)	NA
Meyer	2015		27	22(81)	76.5	Observation	14	7(50)	21.8	0.79 ± 0.59 (0.16)	NA
						Laser	13	0(0)	25.1	1.14 ± 0.70 (0.72)	NA
Cahuzac	2016		12	10(83)	75.1	Observation	5	1(20)	7.7	1.36 ± 0.92 (0.04)	561.60 ± 269.72
						Laser	3	3(100)	7.7	0.50 ± 0.17 (0.32)	494.67 ± 108.09
						Anti-VEGF	4	4(100)	7.7	1.50 ± 0.42 (0.03)	735.50 ± 184.21
PUMCH	2022		14	11(79)	71.6	Observation	5	2(40)	9	0.18 ± 0.20 (0.66)	260.40 ± 89.98
						Laser	4	4(100)	2.75	0.58 ± 0.33 (0.26)	377.50 ± 85.25
						Anti-VEGF	5	1(20)	5.6	1.16 ± 0.78 (0.07)	490.20 ± 210.50
Total			224	NA	72.4 ± 8.9	Observation	124	66(53)	27	0.82 ± 0.75 (0.14)	389.58 ± 175.27
						Laser	80	48(60)	24.6	0.74 ± 0.66 (0.18)	407.50 ± 103.03
						Anti-VEGF	20	9(45)	8.9	1.11 ± 0.63 (0.07)	500.74 ± 177.76

*CMT* Central macular thickness, *NA* Not applicable, *VA* Visual acuity

**Fig. 3:**
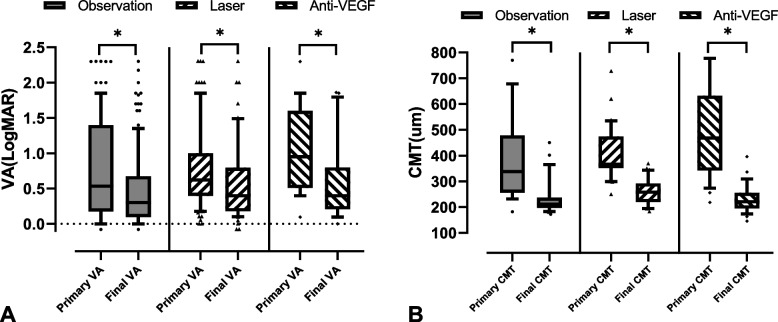
Efficacy of different RAM management. **A** Primary VA (logMAR) changes. **B** CMT (μm) changes

For VA, the primary logMAR VA in the observation group, laser group, and anti-VEGF group were 0.82 ± 0.75, 0.74 ± 0.66, and 1.06 ± 0.63, respectively. The changes in -0.34 ± 0.68(*p* = 0.002), -0.17 ± 0.58(*p* = 0.004), and -0.45 ± 0.62(*p* < 0.001). For CMT, the three groups were 383.87 ± 176.91 μm, 434.33 ± 118.59 μm, and 526.35 ± 187.18 μm, respectively, and changes in CMT improved by -148.26 ± 138.99 µm(*p* < 0.05), -185.61 ± 130.37 µm(*p* < 0.05), and -287.45 ± 171.87 µm(*p* < 0.05) (see Fig. 3).

We performed a subgroup analysis by combining the comparison groups in each study and balancing the interval (m) in each group. For the baseline characteristics of each comparison, the initial VA in each group was balanced, except in the comparison of the laser photocoagulation group and anti-VEGF group. The changes between the initial and final VA between subgroups are shown in Table 3. For the comparison of observation and laser photocoagulation, a total of 192 patients from 9 studies were included. No significant difference was found in this subgroup. For the comparison in the subgroup of observation and anti-VEGF, 42 patients from 3 studies were included. The initial CMT and changes in CMT were significantly different in this group (*p* = 0.013; *p* = 0.005). For the comparison subgroup of laser photocoagulation and anti-VEGF, significant differences were shown in comparison to the initial VA, and there were changes in VA and CMT (*p* = 0.01; *p* = 0.05; *p* = 0.05). More exudative RAM was used in laser photocoagulation than anti-VEGF therapy.

**Table 3:** Subgroup analysis of observation, laser photocoagulation, and anti-VEGF management of RAM

	Observatio (*n =* 124)	Laser Photocoagulatio (*n =* 80)	Anti-VEGF (*n =* 20)	O. vs L.^a^[n(O.) = 112, n(L.) = 80]	O. vs A.^a^[n(O.) = 22, n(L.) = 20]	L. vs A.^a^[n(L.) = 7,n(A.) = 9]
				*p*	*p*	*p*
Interval (m)	26.21 ± 2.74	30.43 ± 3.35	9.43 ± 1.35	0.190	0.800	0.343
Exudative (%)	66(53.2)	48(60.0)	9(45)	0.116	0.863	0.001**
Initial LogMAR VA (mean VA in decimal)	0.82 ± 0.75 (0.15)	0.79 ± 0.60 (0.16)	1.06 ± 0.63 (0.09)	0.132	0.949	0.010**
△VA	-0.34 ± 0.68	-0.17 ± 0.58	-0.45 ± 0.62	0.918	0.733	0.049**
Initial CMT (μm)	383.87 ± 176.91	434.33 ± 118.59	526.35 ± 187.18	0.192	0.013**	0.088
△CMT (μm)	-148.26 ± 138.99	-185.61 ± 130.37	-287.45 ± 171.87	0.261	0.005**	0.047**

*CMT* Central macular thickness, *VA* Visual acuity, *O* Observation, *L* Laser photocoagulation, *A* Anti-VEGF treatment

^a^Head-to-head comparison studies of subgroups

^**^P < 0.05

## Discussion

Our study presented one of the largest cohorts concerning the nonsurgical management of RAM patients. The changes in VA and CMT of RAM were explored for varied treatment options. The overall visual prognosis of RAM was good. We found that observation, laser photocoagulation and anti-VEGF drug treatment can significantly improve vision and CMT. Anti-VEGF therapy was used in patients with worse VA than patients who underwent laser photocoagulation, but achieved better improvement in VA than the laser photocoagulation group. Patients treated with anti-VEGF also had thicker CMT than the observation group, and experienced better changes in CMT than the observation, as well as laser photocoagulation groups. These results may be explained by the features of the three treatments.

Considering the observation method, its effectiveness is due to the self-healing and potential for spontaneous recovery of RAM. In most cases, the course of RAM is benign, and its stages can be summarized as formation, enlargement, thrombosis, fibrosis, and spontaneous involution [7]. In our study, for most cases in the observation group from the PUMCH, RAM was considered to be in stable condition, when VA was greater than 20/32 (0.2, LogMAR) and CMT was lower than 250 µm; thus, observation was applied to these patients, and they gained significant vision improvement. However, some RAM may rupture during the enlargement period, which is responsible for hemorrhagic or exudative complications. The existence of hemorrhage or subretinal fluid may lead to progressive photoreceptor damage, with consequent irreversible visual impairment [8]. Therefore, although observation is the most conservative method for RAM management, it may not be a safe method when there is a risk for RAM rupture or RAM with severe hemorrhage and exudates. Therefore, some recommendations should be given to patients for observation: (1) Regular follow-up (usually once every 3 months) with examinations mainly include visual acuity, fundus examination and OCT examination; (2) Symptom monitoring, seeking medical advice in time when occurring symptoms such as shadow fluttering; (3) Transfer to community doctors or other ophthalmic specialists in order that the patient’s condition can be monitored; (4) Assessing systemic diseases: macroaneurysms are sometimes related to hypertension, hyperlipidemia or other underlying systemic diseases, so patient should see internal medicine doctors.

Laser photocoagulation is the most common therapeutic method for RAM treatment [9, 10]. The laser can be applied either directly or indirectly to the RAM site. Direct laser treatment has been shown to decrease the duration of the lesion, as the laser can be absorbed by the retinal pigment epithelium and the underlying pigmented choroid, resulting in a temperature increase in the adjacent retina that reduces the release of angiogenic factors and inflammatory cytokines [11]. Indirect photocoagulation treatment around a RAM can reduce the oxygen consumption of the retina, consecutively lessen the blood flow in the RAM, and reduce exudation from abnormal surrounding capillaries [12]. Although RAM are sometimes located far from the macula, hemorrhage and exudation may involve the macular zone and impair vision. Laser photocoagulation may inactivate RAM, help absorb hemorrhage, and improve vision. In RAM cases from the PUMCH, laser therapy was mainly used to exude RAM, which was mostly located in the temporal vessels. At present, there are many studies on the advantages and disadvantages of lasers compared with observations with various results. Koinzer et al. [13] found that the mean visual acuity of the observation or laser group for RAM did not improve significantly from baseline to the end of long-term follow-up. Battaglia et al. [10] performed a randomized controlled study and concluded that laser photocoagulation and observation had similar efficacy in the treatment of ruptured RAM in terms of visual gain. Therefore, in our hospital, the decision for laser treatment is generally conservative, mainly considering (1) exudative RAMs, (2) the aneurysm located at a certain distance from the macular area (about 2000um or more), (3) mild to moderate macular edema presented in OCT, and (4) no serious preretinal hemorrhage that blocks the RAM.

However, laser therapy has some complications, including the risk of a vitreous hemorrhage, vessel occlusion, secondary choroidal neovascularization and RAM recurrence. Laser photocoagulation itself can cause RAM rupture and hemorrhage, consequently affecting vision; in addition, laser photocoagulation can cause scars and impair the visual field [14]. Therefore, in choosing whether to use laser treatment, the location of the RAM should be considered, as well as the retinal thickness and visual acuity.

Anti-VEGF drugs have been successfully used to treat RAM in recent years, leading to a decrease in macular edema, although the treatment of RAM with anti-VEGF drugs still belongs to off-label uses [15, 16]. The mechanism of this curative effect is not known but can be hypothesized as follows: (1) VEGF inhibitors can inhibit angiogenesis caused by retinal ischemia in the pathogenesis of RAM, reduce the vascular permeability of VEGF, and decrease RAM hemorrhage and exudation [17]. (2) VEGF inhibitors reduce nitric oxide produced by vascular endothelial cells and promote vasoconstriction, which helps occlude aneurysms and dissolve hemorrhage and exudation. (3) VEGF inhibitors can disrupt the balance between coagulation and fibrinolysis in the cascade reaction of blood coagulation, dissolve focal embolic damage and promote the clearing of subretinal hemorrhage. Pichi et al. [18] reported using intravitreal bevacizumab injections for the treatment of RAM in a prospective uncontrolled study of 37 patients. The researchers found that patients treated with three monthly injections had better visual results. However, Cho et al. [16] found no difference in VA or CMT improvement between intravitreal bevacizumab therapy and observation alone at final follow-up in another retrospective series of 23 patients. They believed the number of eyes included was too small for statistical analysis, but they found a more rapid VA improvement and resolution of macular edema in the bevacizumab-treated group than in the group that took a natural course.

There are no treatment guidelines for RAM. The choice of whether to apply anti-VEGF therapy or laser therapy is controversial in the literature. Cahuzac et al. [19] compared patients receiving these two treatments and found that all patients with subretinal hemorrhage treated with anti-VEGF presented with modest VA gain. They recommended anti-VEGF treatment in patients with serous retinal detachment because it is a safe, technologically mature procedure that enhances visual recovery, although there is a lack of evidence on its long-term effectiveness. In our study, subgroup analysis showed that patients treated with anti-VEGF drugs had lower baseline VA and higher CMT. These patients were characterized by severe development of RAM, which is distinguished by obvious hemorrhage and edema, resulting in a significant decline in vision. For these patients, observation and laser photocoagulation may not directly eliminate subretinal hemorrhage or macular edema. VEGF inhibitors can further reduce macular edema and hemorrhage by reducing the retinal ischemia and leakage caused by RAM rupture to reduce CMT and enhance VA. Therefore, the evidence from our study shows that anti-VEGF drugs are suitable for patients with low baseline vision and high CMT and achieve significant improvements in vision and retinal anatomy.

This study has several limitations. First, RAM is a rare disease, and a limited number of patients was included in the study; therefore, a literature data analysis was added to supplement the patient data, which helped to prevent insufficient results caused by a lack of robust data. Second, this study is a retrospective study. Only controlled studies with sufficient data were included in the study. For studies with missing data or without treatment comparisons, confounding factors were considered to exist, so they were not included. Besides, selection bias may occur, such as loss of patient with good visual acuity, or patients with severe treatment complications who cannot perform post-treatment OCT. Third, most prescription of treatment method in this study was made on specialists’ decision, and different preferences from specialists may affect the study result. Forth, the baseline data of different treatment groups included in the study were not balanced; therefore, the therapeutic effects of observation, laser, and anti-VEGF drugs could not be directly obtained. More data support is needed for detailed subgroup evaluation. Besides, the activity of RAM was assessed by OCT, which is not as accurate as assessed by FFA, which may have some influence in the result.

In conclusion, observation, laser photocoagulation and anti-VEGF therapy are all effective management methods for RAM. Laser photocoagulation is an effective treatment for exudative RAM. Anti-VEGF therapy is intended to better improve patients with severe VA and CMT. All management strategies should consider the unique needs of each patient.

## Supplementary Information

**Additional file 1 .**

## Data Availability

The datasets used and/or analyzed during the current study are available from the corresponding author on reasonable request.

## Abbreviations

RAM: Retinal arterial macroaneurysm
VA: Visual acuity
CMT: Central retinal thickness
FFA: Fundus fluorescein angiography
ICGA: Indocyanine green angiography
