# Supplementary material for: Observation, laser photocoagulation or anti-VEGF therapy in the management of retinal arterial macroaneurysms
Source: BMC Ophthalmol. 2022 Nov 2;22:417. doi: 10.1186/s12886-022-02641-2 (PMC9628022; doi:10.1186/s12886-022-02641-2)
Supplement: Supplementary file 1 — Additional file 1 . [file 12886_2022_2641_MOESM1_ESM.docx]

Search strategy

# Pubmed search strategy

Publication date to January 31st, 2022

Search strategy:

| Search number | Query | Results |
| --- | --- | --- |
| 1 | (“retinal arterial macroaneurysm”[Mesh]) OR (retinal arterial macroaneurysm) | 193 |
| 2 | observation | 3,728,378 |
| 3 | anti-VEGF OR intravitreal OR ranibizumab OR bevacizumab OR aflibercept OR conbercept | 42,938 |
| 4 | laser OR photocoagulation | 369,772 |
| 5 | #2 OR #3 OR #4 | 4,056,167 |
| 6 | #1 AND #5 AND Publication date to January 31st, 2022 | 113 |

**Search detail:**

(("retinal arterial macroaneurysm"[MeSH Terms] OR ("retinal arterial macroaneurysm"[MeSH Terms] OR ("retinal"[All Fields] AND "arterial"[All Fields] AND "macroaneurysm"[All Fields]) OR "retinal arterial macroaneurysm"[All Fields])) AND ("observability"[All Fields] OR "observable"[All Fields] OR "observables"[All Fields] OR "observation"[MeSH Terms] OR "observation"[All Fields] OR "observe"[All Fields] OR "observed"[All Fields] OR "observer"[All Fields] OR "observer s"[All Fields] OR "observers"[All Fields] OR "observes"[All Fields] OR "observing"[All Fields] OR "watchful waiting"[MeSH Terms] OR ("watchful"[All Fields] AND "waiting"[All Fields]) OR "watchful waiting"[All Fields] OR "observations"[All Fields] OR ("anti-VEGF"[All Fields] OR ("intravitral"[All Fields] OR "intravitreal"[All Fields] OR "intravitreally"[All Fields] OR "intravitreous"[All Fields] OR "intravitreously"[All Fields]) OR ("ranibizumab"[MeSH Terms] OR "ranibizumab"[All Fields]) OR ("bevacizumab"[MeSH Terms] OR "bevacizumab"[All Fields] OR "bevacizumab s"[All Fields]) OR ("aflibercept"[Supplementary Concept] OR "aflibercept"[All Fields]) OR ("kh902 fusion protein"[Supplementary Concept] OR "kh902 fusion protein"[All Fields] OR "conbercept"[All Fields])) OR ("laser s"[All Fields] OR "lasers"[MeSH Terms] OR "lasers"[All Fields] OR "laser"[All Fields] OR "lasered"[All Fields] OR "lasering"[All Fields] OR ("light coagulation"[MeSH Terms] OR ("light"[All Fields] AND "coagulation"[All Fields]) OR "light coagulation"[All Fields] OR "photocoagulation"[All Fields] OR "photocoagulations"[All Fields] OR "photocoagulate"[All Fields] OR "photocoagulated"[All Fields] OR "photocoagulating"[All Fields] OR "photocoagulative"[All Fields] OR "photocoagulator"[All Fields] OR "photocoagulators"[All Fields]))))

Filter: date of year: Inception to January 31st, 2022

# EMBASE

Publication date to January 31st, 2022

Database: Embase 1971 to January 31st, 2022

| # | Searches | Results |
| --- | --- | --- |
| 1 | 'retinal arterial macroaneurysm'/exp OR 'retinal arterial macroaneurysm' OR (('retinal'/exp OR retinal) AND arterial AND macroaneurysm) | 1,773 |
| 2 | 'observation'/exp OR observation | 480,638 |
| 3 | 'anti vegf' OR intravitreal OR ranibizumab OR bevacizumab OR aflibercept OR conbercept | 101,317 |
| 4 | laser OR photocoagulation | 421,992 |
| 5 | #2 OR #3 OR #4 | 984,610 |
| 6 | #1 AND #5 AND [1-1-1971]/sd NOT [31-1-2022]/sd | 259 |

Search Detail:

('retinal arterial macroaneurysm'/exp OR 'retinal arterial macroaneurysm' OR (('retinal'/exp OR retinal) AND arterial AND macroaneurysm)) AND ('observation'/exp OR observation OR 'anti vegf' OR intravitreal OR ranibizumab OR bevacizumab OR aflibercept OR conbercept OR laser OR photocoagulation) AND [1-1-1971]/sd NOT [31-1-2022]/sd

# 3 Cochrane

Database: Cochrane Central Register of Controlled Trials（CENTRAL）and Cochrane Database of Systematic Reviews to January 31st, 2022

Search strategy:

| # | Searches | Results |
| --- | --- | --- |
| 1 | retinal arterial microaneurysm:ti,ab,kw | 5 |
| 2 | observation | 48,872 |
| 3 | anti-VEGF OR intravitreal OR ranibizumab OR bevacizumab OR aflibercept OR conbercept | 11,056 |
| 4 | laser OR photocoagulation | 21,858 |
| 5 | #2 OR #3 OR #4 | 79,340 |
| 6 | #1 AND #5 | 5 |

# 4 Web of Science

Database: WOS, MEDLINE, RSCI

Language: Auto

Time range: Inception to 2022.1

| # | Searches | Results |
| --- | --- | --- |
| 1 | TS=(retinal arterial macroaneurysm ) | 197 |
| 2 | TS=(observation ) | 2,057,107 |
| 3 | TS=(anti-VEGF OR intravitreal OR ranibizumab OR bevacizumab OR aflibercept OR conbercept ) | 66,066 |
| 4 | TS=(laser OR photocoagulation ) | 2,030,865 |
| 5 | #4 OR #3 OR #2 | 4,083,924 |
| 6 | #5 AND #1 | 115 |

# 5 Other sources

**ClinicalTrials.gov**

Condition or disease: retinal arterial macroaneurysm

Items: 3
